# Endoleak detection with photon-counting detector CT at various radiation doses after endovascular aneurysm repair: a phantom study

**DOI:** 10.1007/s10554-026-03765-y

**Published:** 2026-08-13

**Authors:** Adrian T. Huber, Gergely Szucs-Farkas, Ludovic G. Perrin, Badis M’Hamdi, Sofia Bespyatova, Andreas Christe, Zsolt Szucs-Farkas

**Affiliations:** 1 Department of Diagnostic, Interventional and Pediatric Radiology, Inselspital, Bern University Hospital, University of Bern, Bern, Switzerland; 2 Department of Radiology and Nuclear Medicine, University Teaching and Research Hospital, Luzerner Kantonsspital, University of Lucerne, Lucerne, Switzerland; 3 University of Bern, Bern, Switzerland; 4 Department of Radiology, Hospital Centre of Biel, Biel/Bienne, Switzerland

**Keywords:** Photon- counting- detector CT, CT Angiography, Abdominal aortic aneurysm, Endoleak, Phantom study

## Abstract

After endovascular aneurysm repair, patients require lifelong CT follow-ups. Photon-counting detector CT (PCD-CT) may improve detection of tiny iodinated structures at low radiation dose. The aim of this experimental study was to find the lowest dose which still provides high detectability of small endoleaks using PCD-CT with 55 keV and 50 keV virtual monoenergetic images (VMIs). An abdominal aneurysm phantom mimicking medium and large patients was scanned at 100%, 50%, 25%, and 12.5% radiation dose using PCD-CT. Besides standard 55 keV images, 50 keV VMI were reconstructed at 25% and 12.5% dose. The ninety-six CT series containing 1728 endoleaks of 2–6 mm were read by three independent observers. Areas under the alternative free-response operational characteristic curve (A_1_) were analysed with the jackknife-method. In the medium phantom, A_1_ for 6 mm endoleaks was similar at 100% and 50% doses (0.93 vs. 0.86; *p* = 0.23), but performance at 50% dose decreased in 4–6 mm endoleaks (A_1_, 0.81 vs. 0.68; *p* = 0.02). In the large phantom, A_1_ decreased at 50% dose both for 6 mm (0.72 vs. 0.42; *p* = 0.003) and for 4–6 mm (0.49 vs. 0.29; *p* = 0.02) endoleaks. Compared with standard reconstruction, 50 keV VMIs did not change A_1_ at 25% dose (0.56 vs. 0.52; *p* = 0.38) and led to worse performance at 12.5% dose (0.30 vs. 0.19; *p* = 0.01). In summary, diagnostic performance for 6 mm endoleaks remained high with 50%-dose reduction using PCD-CT in simulated medium but not in large patients. 50 keV VMIs at 12.5% and 25% dose did not improve endoleak detection.

## Background

Endovascular aneurysm repair (EVAR) of abdominal aortic aneurysms is preferred over open surgery in older and high-risk patients because of its lower short-term mortality [1]. Patients with EVAR undergo regular imaging follow-up to rule out aneurysmal sac growth and endoleaks [2]. The identification and correct classification of endoleaks have a significant impact on the patient’s prognosis, as untreated endoleaks can lead to increased pressure and rupture of the aneurysmal sac [3]. There are no data in the literature regarding the size at which an endoleak is considered as significant; therapeutic decisions are mainly based on the volume increase of the aneurysm and the type of endoleak [4–7]. Computed tomography angiography (CTA) is the most common imaging modality used for follow-up after EVAR. Unfortunately, radiation exposure from repetitive CTA studies can accumulate to potentially harmful amounts during a patient’s lifetime, even if they represent a rather elderly population [8]. Thus, reducing the radiation dose remains important for improving patient safety.

Radiologists are making increasing efforts to reduce radiation exposure after EVAR. In addition to switching to colour duplex US or MR-Angiography, CTA using low tube kilovoltage and replacing non-contrast CT scans with virtual native images from contrast enhanced series acquired with dual-energy technology are the most common methods to achieve this goal with conventional energy-integrating detector CTs (EID-CT) [9–12]. Photon-counting detector CT (PCD-CT) has proven its capability to increase spatial resolution and material decomposition at lower radiation exposure compared to EID-CT in various body regions [13–18]. Based on these findings, it could be possible to reduce the radiation dose while maintaining diagnostic accuracy in patients requiring regular follow-up CTAs after EVAR. Despite some studies on PCD-CT for CTA of the aorta in experimental and clinical settings, no research has yet targeted the potential of PCD-CT in the detection of endoleaks at reduced radiation dose under controlled conditions [19–24]. Additionally, it is not known whether the higher iodine attenuation of endoleaks at lower kiloelectronvolts (keV) could compensate for the expectedly diminished detectability of endoleaks at reduced radiation dose.

In our experimental study, we aimed to determine the lowest radiation dose that still provides a high detectability of small endoleaks in an abdominal aortic aneurysmal phantom using PCD-CT at various simulated body sizes. Additionally, we tested whether virtual monoenergetic images at 50 keV might be useful to improve endoleak detection at reduced dose.

## Methods

### The phantom

**Fig. 1:**
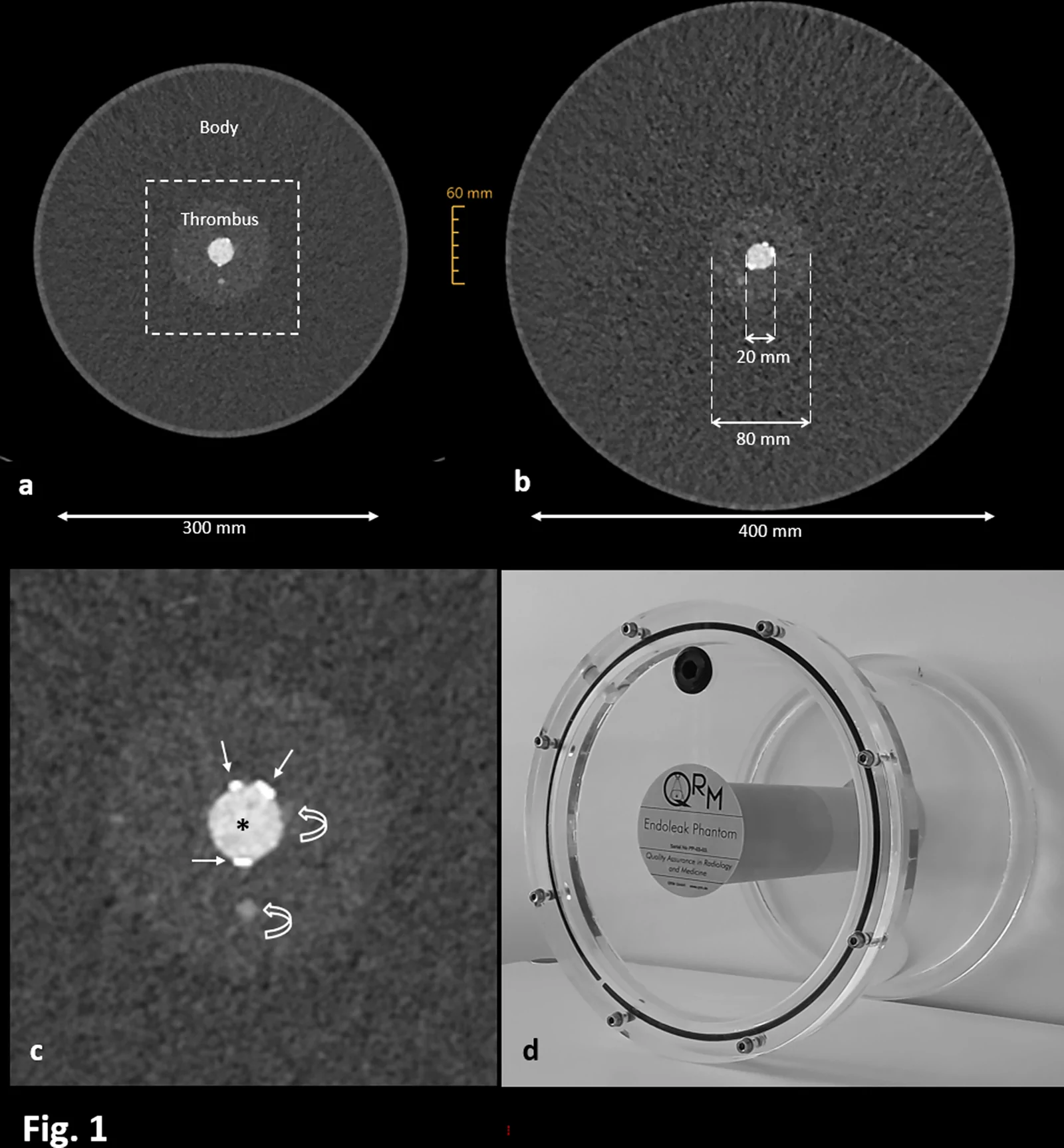
Visualisation of the experimental setting used in the study. Virtual monoenergetic transverse CT images at 55 keV of the aortic aneurysm phantom placed into the 30 cm (*a*) and 40 cm (*b*) water container to mimic medium sized and large patients. Figure *c* shows the magnified central portion of the phantom, marked by dotted rectangle in image *a*. The asterisk signals the aortic lumen and the arrows point at the metallic scaffold of the stent. The curved arrows mark two endoleaks in the simulated thrombus material. Figure *d* shows a photograph of the 30 cm-water container with the cylindrical aneurysm phantom in its central axis

Based on the attenuation measurements in 10 consecutive patients with type II and III-endoleaks after EVAR on CTA in our institution, a custom cylindrical resin phantom mimicking the aneurysmal sac of the abdominal aorta with a length of 21 cm and a diameter of 8 cm was built (QRM, Moehrendorf, Germany). The phantom enclosed a commercial stentgraft (Valiant, Medtronic, Calif.) of 20 mm diameter in its central long axis and multiple 3 mm thick resin pellets, containing anorganic iodine, around the graft to simulate endoleaks. There were 36 endoleaks with three various diameters of 2, 4 and 6 mm, 12 pieces of each, at distances of 0, 5 and 10 mm from the mesh of the stentgraft. In the two halves of the phantom, the attenuation of the leaks was 120 HU and 145 HU at 120 kVp, corresponding to 4.2 and 5.8 mg I/mL water, referred to as low-density and high-density endoleaks, respectively. The graft was filled with a 1:37 mixture of iomeprol (Iomeron 370, Bracco) and tap water. The phantom was placed in water-filled containers with diameters of 30 and 40 cm, mimicking medium- and large patients with estimated body weights of 72–85 and 118–142 kg, respectively [25]; Fig. 1).

## CT examination protocol and image post-processing

The medium water container including the phantom was scanned with a dual-source PCD-CT (Naeotom Alpha, Siemens Healthineers, Erlangen, Germany). Parameters currently used in a standard patient for CTA of the abdominal aorta in our institution were applied: peak kilovoltage, 120 kVp; collimation, 144 × 0.4 mm; rotation time, 0.5s; image quality (IQ) level, 47; quantum iterative reconstruction level, 3; image reconstruction kernel, Bv40. The target volume CT-dose index (CTDI_vol_) was set at 4 mGy which corresponded to the dose level of our currently used EID-CT (Siemens Force, Erlangen, Germany). For the following scans, the radiation dose was reduced to 50%, 25% and 12.5%. This was achieved by changing the IQ-level, which adapted the reference milliampere-second level accordingly.

For the large water container, the CTDI_vol_ was set to 8 mGy for the 100% dose level, corresponding to our EID-CT protocol used in obese patients, and the same dose modulation steps were repeated as described above, leaving all other technical parameters unchanged (Table 1.)

**Table 1:** CT scan parameters and measurements in the aneurysm phantom at various radiation doses

Simulated body size	kVp	IQ	Quality reference mAs	Effective mAs	CTDI_vol_. (mGy)	Dose	Reconstruction	Attenuation Aorta (HU)	Noise (HU)	CNR Aorta
Medium	120	93	51	41	3.98	100%	standard	474 (472–476)	22 (21–22)	19.5 (19.6–20.4)
	120	47	26	21	2.03	50%	standard	472 (470–473)	27 (26–27)	16.3 (15.9–16.7)
	120	23	13	10	1.02	25%	standard	473 (472–477)	33 (33–34)	13.1 (12.7–13.2)
							50 keV	578 (575–580)	37 (36–37)	14.6 (14.4–14.7)
	120	11	6	5	0.47	12.5%	standard	474 (470–479)	44 (43–45)	9.7 (9.5–10.2)
							50 keV	578 (576–585)	50 (48–50)	10.8 (10.6–11.2)
Large	120	75	41	80	7.98	100%	standard	464 (459–469)	29 (29–30)	14.6 (14.2–14.9)
	120	38	21	41	4.09	50%	standard	464 (459–468)	38 (36–39)	11.2 (11.0–11.6)
	120	19	10	19	1.95	25%	standard	432 (427–447)	52 (49–54)	7.7 (7.4–8.2)
							50 keV	530 (524–538)	60 (58–62)	8.2 (8.1–8.6)
	120	9	5	10	0.97	12.5%	standard	419 (414–424)	71 (64–75)	5.4 (5.1–6.1)
							50 keV	475 (473–501)	86 (83–88)	5.4 (5.3–5.5)

Radiation dose in percentage in the seventh column refers to our currently used protocol for CT angiography of the abdominal aorta with our latest generation energy integrating detector CT in average and obese patients. Please note that CTDI_vol_ is twice as high in the large phantom compared to the medium size phantom for the same percentage dose level. Standard image reconstruction corresponded virtual monoenergetic images at 55 keV. Measurements and calculations are not normally distributed and are given as medians with interquartile ranges in parentheses

kVp= peak kilovoltage; IQ= image quality parameter by the CT manufacturer; keV= kiloelectronvolt; mAs= milliamperesecond; CTDI vol., CT dose index volume; mGy= milligray; HU= Hounsfield unit; CNR= contrast to noise ratio

All series were reconstructed at 55 keV and 1 mm slice thickness to mimic standard abdominal CTA protocols at our institution. Additionally, virtual monoenergetic images (VMIs) at 50 keV were generated for the 25% and 12.5% dose levels in both phantoms. This monoenergy level was selected because of promising results on using this level with aortic CTA in the literature [20].

During the post-processing, CT datasets were divided into two series, each containing 18 pieces of either low- or high-density endoleaks. This was done to reduce complexity of the reading. All datasets were rotated by 0°, 90°, 180° and 270°, respectively, and saved on PACS. This resulted in 96 CT series (2 simulated body sizes, 2 phantom-halves, 4 dose levels with standard reconstruction, 2 datasets at 50 keV for the 25% and 12.5% dose-levels, 4 rotations), containing a total of 1728 simulated endoleaks. In this way, the number of analyzable series has been quadrupled but no endoleak in the same localization under the same CT parameters occurred twice. The datasets used and analysed during the current study are available from the corresponding author on reasonable request.

## Quantitative image analysis

Attenuation in the simulated aortic lumen, in the aneurysmal sac, and in the 6 mm endoleaks was measured by the primary investigator with 27 years of CT-experience (ZSF), who did not act as reader. The regions of interest were drawn as large as possible, by carefully avoiding contact with other structures. Image noise was described as standard deviation of the attenuations in the simulated body (water). All measurements were performed in ten different locations at each dose level and monoenergetic reconstruction. The contrast-to-noise ratio (CNR) was calculated for the aorta and for both types of endoleaks as the attenuation difference compared to the aneurysmal sac divided by the image noise.

## Reading session

Three board-certified radiologists with CT experience of 7, 8 and 15 years (SB, BM, LGP), who were blinded to all technical parameters and to the number and position of the endoleaks, independently analysed the 96 CT series in a random order. They marked all possible endoleaks on a schematic drawing of the phantom in the axial plane, along with the slice position and diagnostic confidence (1 = low, 2 = moderate, 3 = high). Readers rated each CT series with respect to image noise (1 = major, 2 = substantial, 3 = moderate, 4 = minor, and 5 = none) and overall quality (1 = bad; 2 = poor; 3 = moderate; 4 = good; and 5 = excellent). The readers were instructed on the reading criteria at the beginning of the session.

Image analysis was performed in ambient light on high-resolution liquid crystal display monitors routinely used for CT image interpretation. As in a real clinical situation, readers were allowed to scroll through the images and adjust magnification and window settings.

### Statistical analysis

Measurements and calculated parameters in the phantom and subjective ratings by the readers were tested for normality with the Shapiro-Wilks-test and compared using nonparametric analysis of variance (Friedman-test). Those analyses were done with the Statistica 7 software package (Statsoft, Tulsa). The level of statistical significance was set at *p* < 0.05. For subjective image quality and subjective image noise the Bonferroni-correction was applied.

Marks by the observers were classified by GSF and ZSF, who did not act as readers, as true positive findings if these were situated within a radius of 5 mm to real endoleaks on the composition plan of the phantom, otherwise as false positive. We set the threshold at 5 mm to accept a mark as true positive, because inaccuracies of 6 mm or more could have left room for an additional “large” 6 mm endoleak between the reader’s mark and the real localization of the simulated endoleak. Data were analysed with the jackknife alternative free response receiver operating characteristics method program (JAFROC Version 4.2.1). The JAFROC1 method includes localization information of multiple marks per dataset and has greater statistical power than other related procedures [26]. Beside the area under the alternative free-response ROC (receiver operating characteristics) curve (A_1_), which represents the probability that lesions are rated higher by observers than non-lesion localizations, i.e. false positives, the mean detection rates over three readers for 2–6 mm, 4–6 mm, and 6 mm endoleaks were analysed separately and compared by using Barnard’s exact test [27]. Krippendorff’s alpha was calculated for the interreader reliability [28].

The preliminary power calculation showed that one-way analysis of variance of 144 endoleaks per CT parameter setting would allow for the detection of 5% difference in sensitivity with a power of 0.849 and an alpha-value of 0.05.

## Results

### Phantom measurements and objective image quality

In the medium phantom, the median aortic attenuation remained stable at different dose levels (medians, 472−474 HU at 55 keV and 578 HU at 50 keV, *p* = 0.992 for all comparisons, Table 1.). In the large phantom, the median aortic attenuation decreased at lower radiation dose levels (464−419 HU at 55 keV, 530−475 HU at 50 keV; p-values between 0.012 and < 0.001). Aortic attenuation was higher at 50 keV than at 55 keV (*p* < 0.001).

**Fig. 2:**
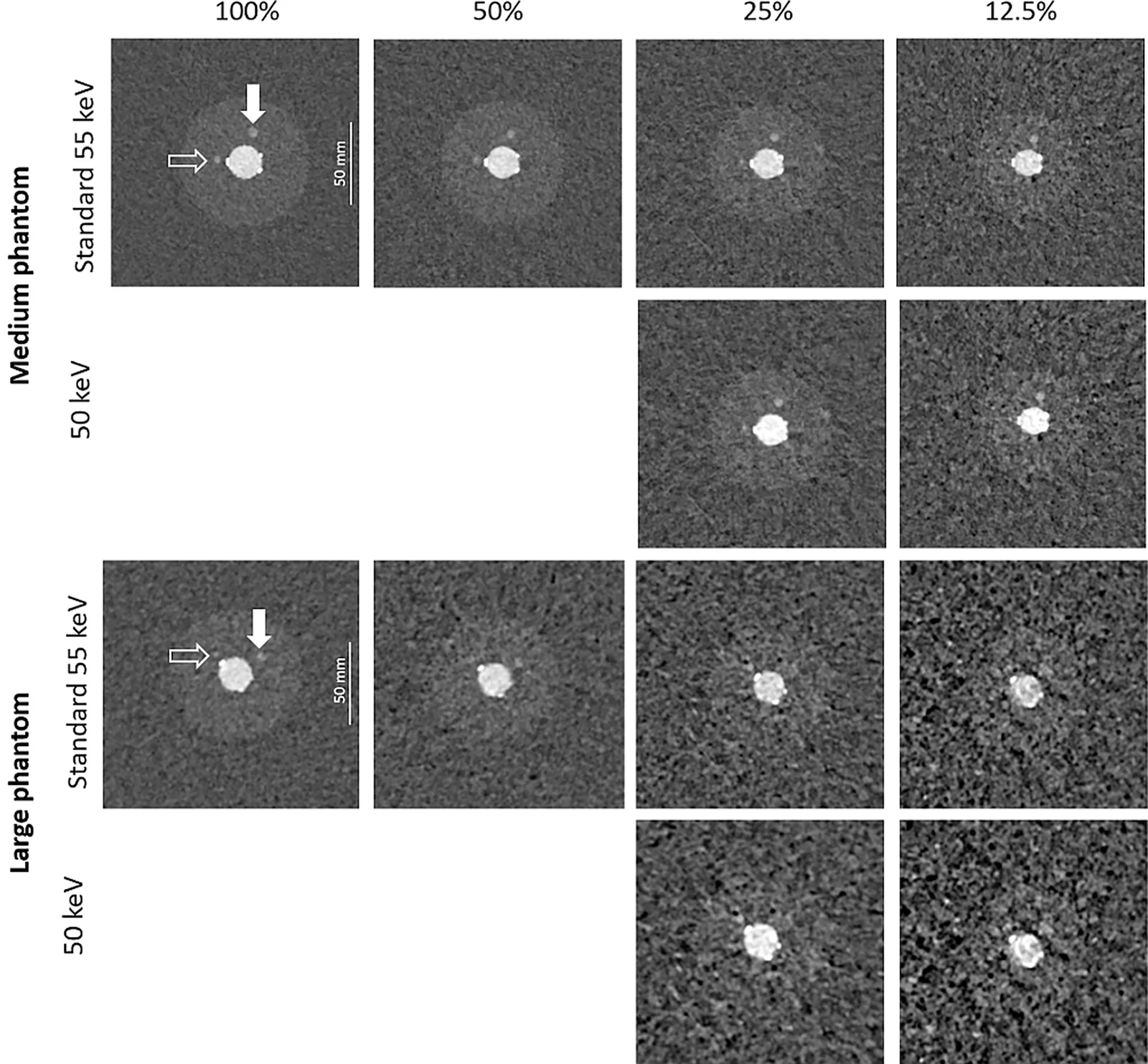
Photon-counting detector CT images at the same level of the abdominal aneurysm phantom with various radiation doses (12.5%-100%). The 100% dose corresponded to a volume CT-dose index of 4 mGy in the medium and 8 mGy in the large phantom. Beside the standard images at 55 keV, additional reconstructions at 50 keV were made for the 25% and 12.5% dose series. In the 100% dose images, the solid arrow points to a 6 mm endoleak and the open arrow to a 4 mm endoleak in the simulated thrombus around the contrasted aortic lumen. The window width was set at 800 HU and the window level at 200 HU for all series. The endoleaks become less discernible with increasing graininess at lower dose, especially in the phantom mimicking large patients. Despite higher iodine density with the 50 keV reconstructions, depiction of endoleaks is not improved compared to the standard 55 keV series

The noise level increased at lower radiation dose and on 50 keV VMIs compared to the standard 55 keV images (p-values between 0.019 and < 0.001). The CNR in the simulated aortic lumen decreased at reduced dose in both phantoms for the standard protocol (*p* < 0.001 for all comparisons). At 50 keV, CNR in the aorta was higher than on the standard 55 keV images in the medium phantom (*p* = 0.002 at 25% and < 0.001 at 12.5% dose), but not in the large phantom (*p* = 0.333 at 25% and 0.998 at 12.5% dose, respectively). CNR in the high density endoleaks (median, 5.9 [IQR, 4.3−7.1] in the medium phantom and 2.6 [IQR, 1.9−3.3] in the large phantom) and in the low-density endoleaks (median, 3.9 [IQR, 3.1−4.9] in the medium phantom and 2.4 [IQR, 1.9−3.0] in the large phantom) showed similar changes as the aortic CNR (Fig. 2).

## Endoleak detection rates

Readers found only 1% (mean detection rate, 8.33/576) of the 2 mm endoleaks across all scanner settings and phantoms (Table 2). For the 4 mm endoleaks in the medium phantom, the mean sensitivity was 33/48 (69%) at 100%, 21/48 (44%) at 50%, and only 11/48 (23%) at 25% dose with standard reconstruction. Mean sensitivity for 4 mm endoleaks were very low in the large phantom with only 1.3−3.3/48 detected endoleaks in the various CT series.

**Table 2:** Number of true positive findings for each reader at various dose levels and reconstructions

Phantom	Dose	Recon		Reader 1 (8 yrs)						Reader 2 (11 yrs)						Reader 3 (7 yrs)						Mean all Readers					
	Diameter (mm)			2		4		6		2		4		6		2		4		6		2		4		6	
		Density		1	2	1	2	1	2	1	2	1	2	1	2	1	2	1	2	1	2	1	2	1	2	1	2
			n	24	24	24	24	24	24	24	24	24	24	24	24	24	24	24	24	24	24	24	24	24	24	24	24
Medium	100%	standard		0	1	14	22	24	24	0	0	14	20	23	24	0	0	11	18	22	24	0	0.3	13	20	23	24
	50%	standard		2	1	9	13	21	24	0	1	10	14	23	23	1	0	7	10	17	23	1	0.7	8.7	12.3	20.3	23.3
	25%	standard		0	0	4	7	20	23	0	1	2	14	20	24	0	0	2	4	13	23	0	0.3	2.7	8.3	17.7	23.3
		50 keV		0	0	4	8	19	24	1	0	4	10	16	24	0	0	2	5	11	20	0.3	0	3.3	7.7	15.3	22.7
	12.5%	standard		0	1	4	3	4	18	0	0	5	2	6	18	0	0	3	1	3	11	0	0.3	4	2	4.3	15.7
		50 keV		0	0	3	2	6	11	0	1	3	4	7	12	0	0	1	2	1	10	0	0.3	2.3	2.7	4.7	11
	*Total (n = 144)*			*2*	*3*	*38*	*55*	*94*	*124*	*1*	*3*	*38*	*64*	*95*	*125*	*1*	*0*	*26*	*40*	*67*	*111*	*1.3*	*2*	*34*	*53*	*85.3*	*120*
Large	100%	standard		1	0	0	3	21	24	0	0	1	3	15	21	0	0	0	1	13	17	0.3	0	0.3	2.3	16.3	20.7
	50%	standard		0	1	1	1	11	14	0	0	0	2	10	15	0	1	0	0	8	9	0	0.7	0.3	1	9.7	12.7
	25%	standard		0	0	1	3	0	8	0	0	3	0	0	10	2	0	1	0	1	8	0.7	0	1.7	1	0.3	8.7
		50 keV		1	1	1	3	0	7	3	0	3	1	1	7	0	0	0	0	1	6	1.3	0.3	1.3	1.3	0.7	6.7
	12.5%	standard		1	0	3	1	0	0	0	0	0	1	2	1	1	0	2	3	0	1	0.7	0	1.7	1.7	0.7	0.7
		50 keV		0	0	1	0	0	1	2	0	0	2	1	1	1	0	0	3	1	0	1	0	0.3	1.7	0.7	0.7
	*Total (n = 144)*			*3*	*2*	*7*	*11*	*32*	*54*	*5*	*0*	*7*	*9*	*29*	*55*	*4*	*1*	*3*	*7*	*24*	*41*	*4*	*1*	*5.7*	*9*	*28.3*	*50*

CT-experience in years is provided for each reader in the second row. Code 1 in the fourth row denotes low density endoleaks and code 2 is for high density leaks. Recon= image reconstruction at standard 55 keV or 50 keV. All values are numbers of true positive findings. For each cell, the number of endoleaks to find was 24, summing up to a total of 144 leaks per size and density for 6 CT series in each phantom

**Fig. 3:**
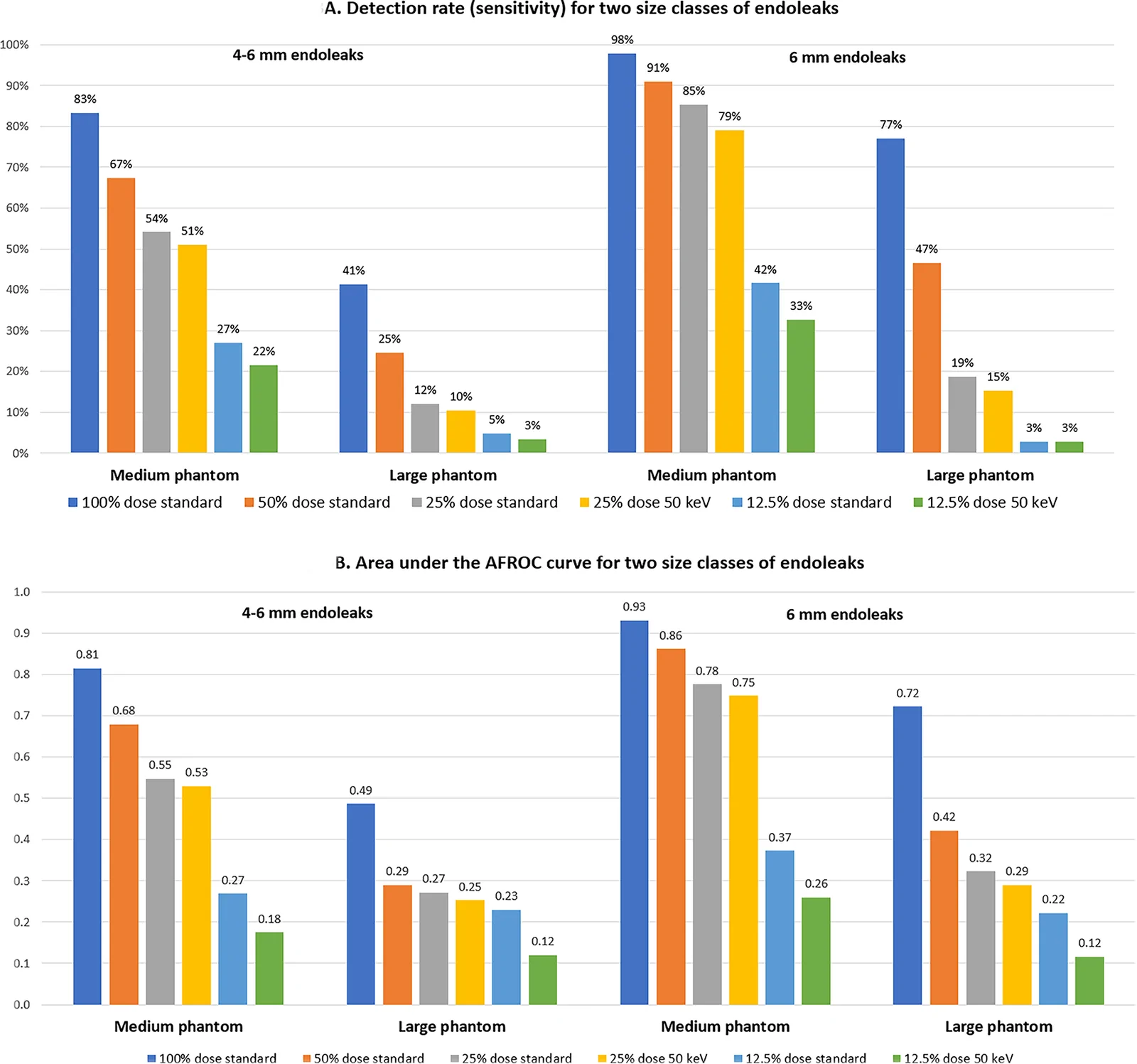
Bar charts including numerical values for the sensitivity (*a*) and A_1_ (*b*), the latter was used to describe readers’ performance for endoleak detection with various CT protocols. All charts show data for endoleaks with probable clinical significance (size, 4–6 mm and 6 mm) in the medium and large phantom separately

For the 6 mm endoleaks in the medium phantom, the mean sensitivity was 47/48 (98%) at 100% and 43.7/48 (91%) at 50% dose (*p* = 0.322). A reduction to 25% radiation dose lowered detection rate to 41/48 (85%) endoleaks (*p* = 0.045). In the large phantom, readers found 37/48 (76%) 6 mm endoleaks at 100% radiation dose, 22.3/48 (47%) endoleaks at 50%, and 9/48 (15%) endoleaks at 25% radiation dose with standard 55 keV reconstruction (*p* < 0.01 to < 0.001; Fig. 3a.).

If 4–6 mm endoleaks were pooled together, average detection rate dropped from 80/96 (83%) to 64.7/96 (67%; *p* = 0.02) between 100% and 50% radiation dose, and to 52/96 (54%; *p* < 0.001) at 25% dose with standard 55 keV reconstruction. This was accentuated in the large phantom with mean detection rates of 39.7/96 (41%) at 100%, 23.7/96 (25%) at 50%, and 11.7/96 (12%) at 25% dose (*p* < 0.001; Table 2; Fig. 3a). There was no difference in detection rates between standard 55 keV and 50 keV VMIs at 25% and 12.5% dose (p-values between 0.489 and 0.869, Table 3.).

**Table 3:**
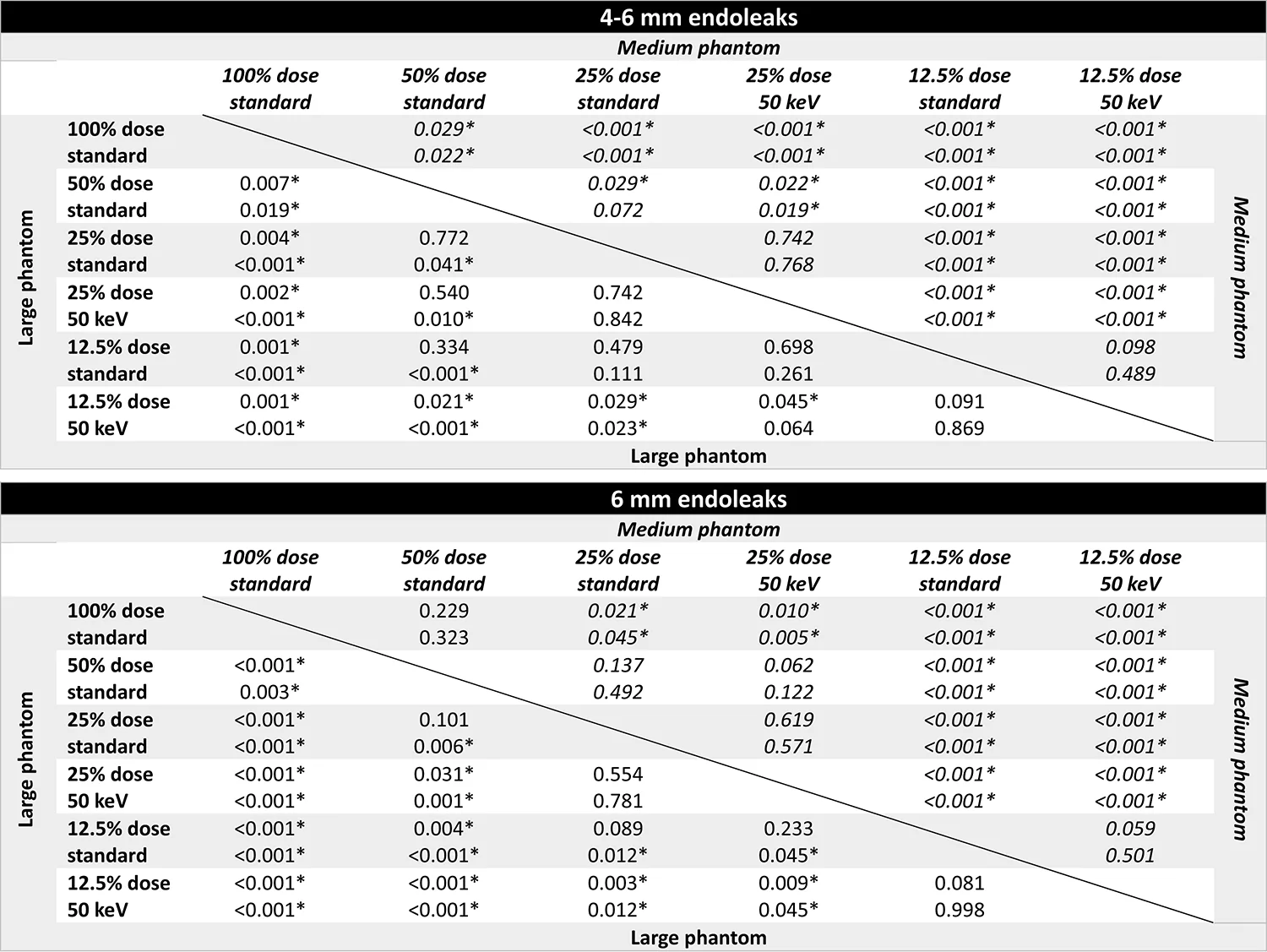
Comparison of mean readers’ performance for the detection of endoleaks in two size classes

The table shows separate analyses for 4-6 mm endoleaks and for 6 mm endoleaks only. Data from the large phantom are in the left lower corner, those from the medium phantom are in the right upper corner in italic. The first p-value in each cell is derived from the JAFROC1-statistics comparing areas under the alternative free-response ROC curve as showed in Figure 3b. The second p-value in the cells is from Barnard’s exact test comparing the detection rates shown in Figure 3a. Significant results are marked with asterisk.

Krippendorff’s alpha over all endoleaks was 0.698, indicating moderate interreader agreement.

### False positives and A_1_

Readers marked the most false positive findings in the medium sized phantom at 12.5% dose level with 50 keV, the difference was significant from all other settings (p-values 0.023 to < 0.001) except for the 12.5% dose level with standard reconstruction (*p* = 0.06; Table 4). Compared to the standard 100% radiation dose, the area under the alternative free-response ROC curve A_1_ was not significantly lower at 50% radiation dose for 6 mm endoleaks in the medium phantom (0.93 vs.0.86; *p* = 0.229), but decreased to 0.78 for 25% radiation dose with standard 55 keV reconstruction (*p* = 0.021), as shown in Table 3; Fig. 3b. In contrast, A_1_ significantly decreased for 6 mm endoleaks in the large phantom from 100% to 50% and 25% radiation dose (0.72 vs. 0.42 and 0.32; *p* < 0.001).

**Table 4:** Summary of readers’ ratings in the CT series

Phantom	Dose	Reconstruction	No. FP	Diagnostic confidence	Subjective image quality	Subjective image noise
Medium	100%	standard	5.3	2.4 [2.3–2.5]	4.6 [4.2–4.9]	3.9 [3.4–4.4]
	50%	standard	6.3	2.0 [1.9–2.2]	4.1 [3.8–4.5]	3.3 [3.0-3.7]
	25%	standard	7.7	1.9 [1.8–2.1]	3.4 [2.9-4.0]	3.3 [2.8–3.7]
		50 keV	10	2.0 [1.9–2.2]	3.3 [3.0-3.7]	2.7 [2.1–3.3]
	12.5%	standard	13	1.5 [1.3–1.7]	2.5 [2.2–2.8]	2.3 [1.9–2.6]
		50 keV	21.7	1.7 [1.5–1.9]	2.5 [2.0-2.9]	2.5 [2.0–3.0]
Large	100%	standard	6	1.7 [1.5–1.9]	3.4 [2.9–3.9]	2.9 [2.7–3.1]
	50%	standard	9.3	1.4 [1.3–1.6]	2.7 [2.3–3.1]	2.8 [2.4–3.2]
	25%	standard	9.3	1.6 [1.2-2.0]	2.4 [2.1–2.7]	2.2 [2.0-2.4]
		50 keV	12	1.5 [1.2–1.9]	2.2 [1.8–2.6]	1.9 [1.7–2.1]
	12.5%	standard	9	1.1 [0.9–1.37]	1.8 [1.5–2.1]	1.7 [1.4-2.0]
		50 keV	10.3	1 [1.0–1.0]	1.5 [1.3–1.7]	1.7 [1.4-2.0]

All data are means of 3 readers. The 95% confidence intervals are provided in brackets for diagnostic confidence, subjective image quality and image noise. This presentation was chosen instead of medians and interquartile ranges to better discern differences in the results between the various CT series. The fourth column contains the total number of false positive (FP) findings. Diagnostic confidence was rated on a 3-grade scale, higher numbers correspond to higher confidence levels. A 5-grade scale was used for the subjective image quality and image noise, higher rates denote better quality and less noise

If 4–6 mm endoleaks were analysed together, readers showed the best performance at 100% dose in the both phantoms, with a decrease of A_1_ from 0.81 to 0.68 and 0.55 at 50% and 25% dose (p-values between 0.029 and < 0.001) in the medium phantom and from 0.49 to 0.29 and 0.27 (p-values between 0.007 and < 0.001) in the large phantom.

In the pooled data of both phantoms, 50 keV VMIs yielded similar reader performance as the standard 55 keV protocol at 25% dose for all endoleaks (*p* = 0.749), for the 4–6 mm (*p* = 0.578) and 6 mm endoleaks (*p* = 0.376). At the 12.5% dose level, A_1_ was even lower with 50 keV VMIs than with standard 55 keV reconstruction for 4–6 mm (*p* = 0.019) and for 6 mm endoleaks (*p* = 0.012.).

### Diagnostic confidence and subjective image quality

The mean diagnostic confidence of readers over all CT protocols was higher in the medium (mean, 2.0; 95%CI, 1.94−2.01) than in the large phantom (mean, 1.49; 95%CI, 1.39−1.59; *p* < 0.001). In the medium phantom, readers were more confident at the 100% dose level than at lower dose (p-values between 0.006 and < 0.001, Table 4). The confidence with standard 55 keV and 50 keV VMIs was similar at 25% (*p* = 0.998) and 12.5% dose (*p* = 0.978). In the large phantom, differences in mean diagnostic confidence at various dose levels and keV-reconstructions did not reach statistical significance (p-values between 0.072 and 0.995).

Observers gave the best scores for image quality in both phantoms at the 100% dose level, with significant differences compared to all other settings (*p* < 0.001 after Bonferroni-correction) except for the 50% dose level in the medium phantom (*p* = 0.994; Table 3). Mean quality ratings for 50 keV VMIs and standard 55 keV images were comparable (*p* = 0.989). Image noise at the 100% dose level was rated similarly as for 50% and 25% dose levels with standard reconstruction (p-values 0.123 to 0.993).

## Discussion

This phantom study using PCD-CT shows that detectability reaches 91% for 6 mm endoleaks in simulated medium-sized patients, even after 50% radiation dose reduction, but can significantly deteriorate in the large phantom and for smaller endoleaks. Monoenergetic reconstructions at 50 keV did not improve diagnostic accuracy or reader confidence at 25% and 12.5% radiation dose levels.

In our investigation, we analysed different clinical scenarios and calculated diagnostic performance for various endoleak sizes. We selected this approach even knowing that there is no established threshold above which endoleaks require treatment to prevent aneurysmal rupture.

Based on our findings, radiation dose levels must be adapted to the scanned volume size and the intended detection level of small endoleaks. If the focus is on 6 mm and larger endoleaks in an average patient, radiation dose may be reduced from 100% to 50% using PCD-CT, without significant loss of diagnostic performance, and even to 25% if a slightly lower performance is acceptable. The data from our phantom experiment suggest that in obese patients the dose probably cannot be lowered without deteriorating the diagnostic performance, even for larger 6 mm endoleaks. When the threshold diameter of endoleaks is set at 4 mm, no reduction of radiation dose is advisable, neither in medium-sized nor large objects, because of the diagnostic penalty. Notably, the diagnosis of 2 mm endoleaks was very challenging at all radiation dose levels in both simulated patient sizes. Of course, these findings must be validated in real patients.

The detection rates of endoleaks and A_1_ as a substitute for diagnostic performance showed very similar trends. These values at 100% radiation dose in the large phantom were comparable to those at 25% radiation dose in the medium phantom. The lower detectability of endoleaks in large patients has already been described and may be attributed to the lower spatial resolution at larger field-of-view and to the deteriorated CNR in overweight subjects [29; 30]. A recent experimental study showed that the ultra-high-resolution mode of PCD-CT can facilitate endoleak detection in obese subjects [23].

As expected, the 50 keV VMIs provided a higher iodine attenuation than the standard protocol using 55 keV for each simulated body size. However, 50 keV improved the CNR only in the medium but not in the large phantom, as the higher iodine density could not compensate for the increased image noise. Decreasing iodine density at a lower dose in the large phantom indicated that beam hardening, which is known from studies with EID-CTs, was not completely eliminated by PCD-CT [31].

It should be noted that the dose levels tested in this experiment were rather low, with a CTDI_vol_ of 4 and 8 mGy for simulated medium and large patients for 100%, however, these doses correspond to the target dose for abdominal CTA with a latest generation EID-CT in our institution. For comparison, doses of 10.8 and 20.6 mGy were applied for the same patient sizes at 120 kVp in a phantom study using an older EID-CT for endoleak detection in 2009 [30]. We think that an increase in the radiation dose to 10–12 mGy in the large phantom and its effect on endoleak detection with PCD-CT may be discussed and analysed in subsequent follow-up investigations.

We investigated whether VMIs at lower keV might have a positive effect on the detectability of small iodinated endoleaks at very low radiation doses. Although a recent publication reported good endoleak detection with 50 keV VMIs using EID-CT in 125 patients after EVAR, 50 keV VMIs at reduced dose could not improve any of the investigated diagnostic or quality criteria in our current experiment using PCD-CT [9]. The possible causes for the discrepancies between the results of the two studies are unclear, but differences in the characteristics of the endoleaks (size and density), CT detector technologies, and applied radiation doses may play a role. Our finding is in line with a current experimental paper investigating a broad range of keVs using PCD-CT and suggesting that large patients may need 55–60 keV for optimal endoleak detection [23].

Our study had several limitations. Although our phantom provided a powerful ground truth to test the diagnostic performance, it did not reflect the circumstances of a real-life scenario with breathing, motion, and pulsation artefacts. We simulated stationary conditions and compared only one virtual monoenergy level using our standard protocol. Testing of multiple VMIs at normal dose with PCD-CT has been previously assessed and thus was not an aim in this experiment [23]. A direct comparison of the diagnostic performance with a conventional EID-CT unit was not our goal either. This approach or the evaluation of diagnostic performance at various radiation dose levels at 55 and 60 keV in combination with the ultra-high-resolution mode with PCD-CT would be interesting topics for future investigations. 

We also note that while the observers were experienced, the number of readers was relatively low. More readers with a broader spectrum of experience may yield different results. Although our data must be validated in patients, the data could possibly be used not only to optimise CTA after EVAR, but also to improve PCD-CTA protocols for detecting other small extravasations such as gastrointestinal bleedings. We also think that a consensus on the target size of endoleaks to be detected should be established to facilitate the selection of optimal CT scan parameters.

In conclusion, the detection rate of 6 mm endoleaks with photon-counting-detector CT remained high with a reduced 50% radiation dose level in simulated medium-sized patients in this phantom experiment. However, endoleak detection significantly deteriorated in mimicked large patients and for 4 mm endoleaks at reduced dose. Very small endoleaks measuring 2 mm in diameter had very poor detection rates at all investigated radiation doses. We propose further investigations to determine the best combination of radiation dose and monoenergy level for this clinical task.

## Data Availability

The datasets used and/or analysed during the current study are available from the corresponding author on reasonable request.

## Abbreviations

CNR: Contrast-to-noise ratio
CTA: CT angiography
EID-CT: Energy integrating detector CT
EVAR: Endovascular aneurysm repair
keV: Kiloelectronvolt
JAFROC: Jackknife alternative free-response operational characteristics
VMI: Virtual monoenergetic image
PCD-CT: Photon-counting detector CT
